# ARHGAP25 Inhibits Pancreatic Adenocarcinoma Growth by Suppressing Glycolysis via AKT/mTOR Pathway

**DOI:** 10.7150/ijbs.55919

**Published:** 2021-04-24

**Authors:** Wen-Kuan Huang, Yi Chen, Huafang Su, Tung-Ying Chen, Jiwei Gao, Yaxuan Liu, Chun-Nan Yeh, Shuijie Li

**Affiliations:** 1 Division of Hematology-Oncology, Department of Internal Medicine, Chang Gung Memorial Hospital, Linkou; Chang Gung University College of Medicine, 333, Taoyuan, Taiwan.; 2 Department of Oncology‐Pathology, Karolinska Institutet, BioClinicum J6:30, Karolinska University Hospital, SE-17164 Solna, Sweden.; 3 Department of Radiation and Medical Oncology, the First Affiliated Hospital of Wenzhou Medical University, No.2 Fuxue Lane, Wenzhou 325000, Zhejiang, China.; 4 Department of Pathology, MacKay Memorial Hospital, Taipei, Taiwan.; 5 Department of Surgery and Pancreatic Cancer Team, Chang Gung Memorial Hospital, Linkou; Chang Gung University College of Medicine, 333, Taoyuan, Taiwan.; 6 Department of Microbiology, Tumor and Cell Biology, Karolinska Institutet, SE-17177 Stockholm, Sweden.

**Keywords:** pancreatic adenocarcinoma, ARHGAP25, AKT/mTOR signaling, glycolysis, proliferation.

## Abstract

Increasing evidence reveals that the Rho GTPase-activating protein is a crucial negative regulator of Rho family GTPase involved in tumorigenesis. The Rho GTPase-activating protein 25 (ARHGAP25) has been shown to specifically inactivate the Rho family GTPase Rac1, which plays an important role in pancreatic adenocarcinoma (PAAD) progression. Therefore, here we aimed to clarify the expression and functional role of ARHGAP25 in PAAD. The ARHGAP25 expression was lower in PAAD tissues than that in normal pancreatic tissues based on bioinformatics analysis and immunohistochemistry staining. Overexpression of ARHGAP25 inhibited cell growth of AsPC-1 human pancreatic cancer cells in vitro, while opposite results were observed in BxPC-3 human pancreatic cancer cells with ARHGAP25 knockdown. Consistently, in vivo tumorigenicity assays also confirmed that ARHGAP25 overexpression suppressed tumor growth. Mechanically, overexpression of ARHGAP25 inactivated AKT/mTOR signaling pathway by regulating Rac1/PAK1 signaling, which was in line with the results from the Gene set enrichment analysis on The Cancer Genome Atlas dataset. Furthermore, we found that ARHGAP25 reduced HIF-1α-mediated glycolysis in PAAD cells. Treatment with PF-04691502, a dual PI3K/mTOR inhibitor, hampered the increased cell growth and glycolysis due to ARHGAP25 knockdown in PAAD cells. Altogether, these results conclude that ARHGAP25 acts as a tumor suppressor by inhibiting the AKT/mTOR signaling pathway, which might provide a therapeutic target for PAAD.

## INTRODUCTION

Pancreatic adenocarcinoma (PAAD) is the 7th leading cause of cancer-related death worldwide 1. By 2030, PAAD is predicted to surpass breast, prostate, and colorectal cancers, becoming the second leading cause of cancer-related deaths in the United States 2. While radical resection is the mainstay of curative treatment, more than 80% of patients were diagnosed with an advanced unresectable stage. Despite recent advances in multimodality therapy, the 5-year survival of pancreatic cancer for all stages is 9% 3. Moreover, the efficacy of personalized targeted therapy and immunotherapy for advanced PAAD is limited 4. Therefore, there is an urgent need to gain insights into the molecular mechanisms of pancreatic cancer development, which will help identify potential targets for novel therapeutic strategies.

The Rho GTPase family, primarily Rho, Rac, and Cdc42, plays an oncogenic role in cancer development by regulating cell adhesion and motility, transcriptional expression, and cell cycle progression 5. The Rho GTPase activating protein (RhoGAP) family inactivates Rho GTPases by stimulating GTP-hydrolysis activity to the inactive GDP-bound state. Several RhoGAPs, including deleted in liver cancer 1 (DLC1) 6, ARHGAP24 7, ARHGAP26 8, and ARHGAP10 9, appear to function as tumor suppressors. However, a few ARHGAPs, such as ARHGAP11A and ARHGAP5, act as oncoproteins to promote cancer cell proliferation, invasion, and metastasis 10-12. The role of most RhoGAPs in cancer remains elusive and requires further study.

ARHGAP25 belongs to one subfamily of RhoGAPs, including ARHGAP24 and ARHGAP22 based on the homology 13. Only a few studies reported the significance of ARHGAP25 in cancer. ARHGAP25 has been found to suppress rhabdomyosarcoma cell invasion 14. ARHGAP25 has also been shown to inhibit lung cancer cell development through Wnt/β-catenin signaling pathway 15. However, the biological functions of ARHGAP25 in pancreatic cancer are poorly understood. Moreover, ARHGAP25 specifically catalyzed GTP hydrolysis on Rac1, which has been shown to play an essential role in the initiation and progression of PAAD 16-17. Therefore, investigations into the role of ARHGAP25 will gain a better understanding of the molecular mechanisms underlying PAAD progression.

In this study, we found that ARHGAP25 was downregulated in PAAD. Upregulation of ARHGAP25 inhibited cancer cell growth in vivo and in vitro by suppressing glycolysis through inhibition of AKT/mTOR signaling pathway. These data indicated that ARHGAP25 acts as a tumor suppressor in PAAD cells.

## MATERIAL and METHODS

### TCGA and GEO datasets

Gene expression of ARHGAP25 of PAAD and normal tissues were obtained from publicly available datasets, including The Cancer Genome Atlas (TCGA) PAAD database and Gene Expression Omnibus (GEO) datasets (accession number: GSE15471 and GSE16515). For TCGA dataset and GEO datasets, mRNA expression data were downloaded from the UCSC Xena browser (https://xenabrowser.net) and the Oncomine database (https://www.oncomine.org), respectively.

### Cell culture

One human normal pancreatic duct epithelial cell line (HPNE) and five pancreatic cancer cell lines (AsPC-1, PANC-1, BxPC-3, SW1990, and Capan-1) were purchased from the American Type Culture Collection (ATCC). HPNE, PANC-1, SW1990, and Capan-1 cells were cultured in Dulbecco's modified Eagle's medium (DMEM, Gibco, USA) supplemented with 10% fetal bovine serum (FBS, Gibco), penicillin (100 units/mL), and streptomycin (100 µg/ml). AsPC-1 and BxPC-3 cells were cultured in RPMI 1640 supplemented with 10% fetal bovine serum penicillin (100 units/mL), and streptomycin (100 µg/ml). Cells were incubated in a humidified atmosphere of 5% CO2 at a constant 37°C.

### Human tissue microarray (TMA) and immunohistochemistry (IHC)

A commercially available TMA (HPanA150Su-01) was obtained from Shanghai Outdo Biotech Company, containing 90 cases of pancreatic tumor tissues (60 of 90 had their adjacent non-tumor tissues) for IHC staining. Briefly, after deparaffinization, rehydration, and heat-induced antigen retrieval, the tissue section was blocked with hydrogen peroxide (Thermo) and bovine serum albumin (Thermo). Then this section was incubated with ARHGAP25 (Thermo, PA557238, 1:200) for 40 min, and developed with DAB solution (Thermo). The percentage of positively stained cells was calculated and scored as 0 (negative), 1 (1-5% positive), 2 (5-50% positive) and 3 (51-100% positive).

### Gene set enrichment analysis (GSEA)

GSEA was carried out using the GSEA software (http://www.broadinstitute.org/gsea). We analyzed TCGA PAAD dataset for comparing differential gene expression between the ARHGAP25-high and -low groups divided by the median level of ARHGAP25 expression through the MSigDB C2 CP collection (Canonical pathways gene set).

### Lentiviral production and infection

Lentiviral particles were produced by transfecting HEK293T cells with packaging vector (pSPAX2), envelope vector (pMD2.G), and pLKO.1 cloning vector (Addgene) containing short hairpin RNA (shRNA) targeting ARHGAP25 or pLVX-Puro cloning vector (Clonetech) expressing ARHGAP25 using Lipofectamine 2000 (Thermo Fisher Scientific). Inserts of plasmid constructs were confirmed by DNA sequencing. The shRNA sequences and the primers for the coding sequence of ARHGAP25 were listed in Supplemental Table S1. The supernatants were collected at 48 h after transfection, filtered (0.45 μm), and stored at -80°C. BxPC-3 and AsPC-1 cells were plated in 6-well plates at 5 x 10^5^ cells/well until 70% confluence. BxPC-3 cells were transduced with lentiviral particles packed with pLKO.1 shRNA constructs against ARHGAP25 or the negative control (sh-NC) at the multiplicity of infections (MOI) of 5 in the presence of 5 μg/ml of polybrene for 24 h. AsPC-1 cells were infected with the lentiviral vector pLVX-Puro encoding ARHGAP25 or the empty vector (pLVX-Puro) as the negative control at the MOI of 5 along with 5 μg/mL of polybrene. After 24 h, the virus-containing medium was replaced with complete fresh medium. ARHGAP25 expression was assessed using quantitative real-time reverse transcription-polymerase chain reaction (qRT-PCR) and Western blot 48 h after infection.

### RNA extraction and qRT-PCR analysis

Total RNA from cultured cells was extracted using Trizol Reagent (Invitrogen). First-strand cDNA was synthesized using M-MLV Reverse Transcriptase (Fermentas). The qRT-PCR reaction was performed using Maxima SYBR Green Rox qPCR master mix (Thermo Fisher Scientific) on the ABI PRISM 7300 Sequence Detection System (Applied Biosystems). The PCR primers used in this study were as follows: hsa-ARHGAP25: forward, 5'-CTGGCTACTGTGATTGGTG-3', and reverse, 5'-TTGTATCAGAGTCGCTTGTC-3'; hsa-GAPDH: forward, 5'-GGATTGTCTGGCAGTAGCC-3', and reverse, 5'-ATTGTGAAAGGCAGGGAG -3'.

### Western blot analysis

Total protein was extracted using Pierce RIPA buffer (Thermo Fisher Scientific) and quantified using the BCA assay (Thermo Fisher Scientific). Equal amounts of protein were separated by 10% or 12% sodium dodecyl sulfatepolyacrylamide gel electrophoresis (SDS-PAGE) and subsequently transferred onto nitrocellulose membranes (Millipore). After blocking with 5% nonfat milk in Tris-buffered saline with 0.1% Tween-20 at room temperature for 1 h, membranes were incubated with primary antibodies overnight at 4°C and corresponding secondary antibodies for 1 h at room temperature. The protein bands were visualized with Immobilon Western Chemiluminescent HRP Substrate (Millipore). The antibodies included ARHGAP25 (Abcam, ab192020, 1:1000), HIF-1α (Abcam, ab51608, 1:2000), PKM2 (Abcam, ab137852, 1:500), LDHA (Abcam, ab125683, 1:1000), AKT (Abcam, ab18785, 1:500), p-AKT (Abcam, ab38449, 1:1000), mTOR (Abcam, ab134903, 1:10000), p-mTOR (Abcam, ab109268, 1:5000), PAK1 (Cell Signaling Technology, #2602), p-PAK1 (Cell Signaling Technology, #2601), GAPDH (Cell Signaling Technology, #5174, 1:2000), horseradish peroxidase (HRP) conjugated goat anti-rabbit IgG (Invitrogen, 65-6120, 1:3000), and HRP-conjugated goat anti-mouse IgG (Invitrogen, 62-6520, 1:10000).

### Cell counting kit (CCK-8) assay

Cell proliferation was measured using the cell counting kit-8 (CCK-8) kit (SAB biotech). Briefly, 3000 cells/well were seeded in 96-well plates two days after infection. After incubation for 0, 24, 48 and 72 h, 10 μL CCK-8 solution mixed with fresh 100 μL culture medium was added to each well and incubated at 37°C for 1 h. The absorbance was measured at 450nm. Gemcitabine and 5-Fluorouracil for cytotoxicity assay were purchased from Selleck Chemicals. PF-04691502 (Selleck) was used for AKT-mTOR pathway inhibition.

### Glucose uptake, lactate, and ATP measurement

After lentiviral transduction as indicated, 5 x 10^5^ AsPC-1 and BxPC-3 cells were seeded into six‐well plates and incubated for 48 hours. Glucose uptake and lactate levels in culture supernatants were quantified using the 2-NBDG Glucose Uptake Assay (BioVision) and the Lactate Assay kit (BioVision), respectively. Intracellular ATP content was determined using an ATP Assay Kit (Roche) according to the manufacturer's instructions.

### Mouse Xenograft model

Female Balb/c nude mice (4-6 weeks old, 20g +/-2 g) were purchased from the Chinese Science Academy (Shanghai) and kept under specific pathogen-free conditions. All experimental procedures were performed according to the protocols approved by the Laboratory Animal Ethics Committee of Wenzhou Medical University and Laboratory Animal Centre of Wenzhou Medical University (wydw 2020-0847). 4 x 10^6^ BxPC-3 cells transduced with sh-NC or sh-ARHGAP25 and 4 x 10^6^ AsPC-1 cells transduced with empty vector (NC) or ARHGAP25 overexpressing vector (OE) were injected subcutaneously into left axilla of each nude mouse (6 mice per group). Once tumors became palpable, growing xenografts were measured with a caliper every three days. The volume of xenografts was calculated using the formula: V (mm^3^) = ½ x length x width^2^. All mice were euthanized and sacrificed 33 days after inoculation. The xenografts were harvested, imaged and weighed. The tumor tissues were used for subsequent immunofluorescence and western blot analysis.

### Immunofluorescence

Slides of xenografts were deparaffinized, rehydrated through an alcohol series, and subjected to heat-induced antigen retrieval using 0.01 M sodium citrate buffer solution (pH=6.0). Sections were incubated with primary antibodies against Ki67 (Abcam, ab16667; 1:100) and PCNA (Abcam, ab92552; 1:100) overnight at 4°C. After washing, sections were incubated with the Alexa Fluor 488 anti‐rabbit secondary antibody (Invitrogen, A-11008, 1:1000) for 1 h at room temperature. The nuclei were stained using DAPI (Sigma), and immunofluorescence images were captured using a fluorescence microscope (Nikon).

### Statistical analysis

Data are presented as the mean ± SD of at least three independent experiments. GraphPad Prism 8 (GraphPad Software) was used to generate graphs and statistical analyses. Student's t-test with or without Welch's correction and ANOVA with post hoc Dunnet's test were used to compare the means of two and more than two groups, respectively. The correlation between ARHGAP25 expression and clinicopathological characteristics was calculated by the chi-square test. The difference of ARHGAP25 expression in normal and tumor tissues was analyzed using the Mann-Whitney U test and chi-square test for transcriptome data and IHC staining scores. Probability (P) value < 0.05 was indicated as statistically significant.

## RESULTS

### ARHGAP25 expression is downregulated in PAAD

**Figure 1:**
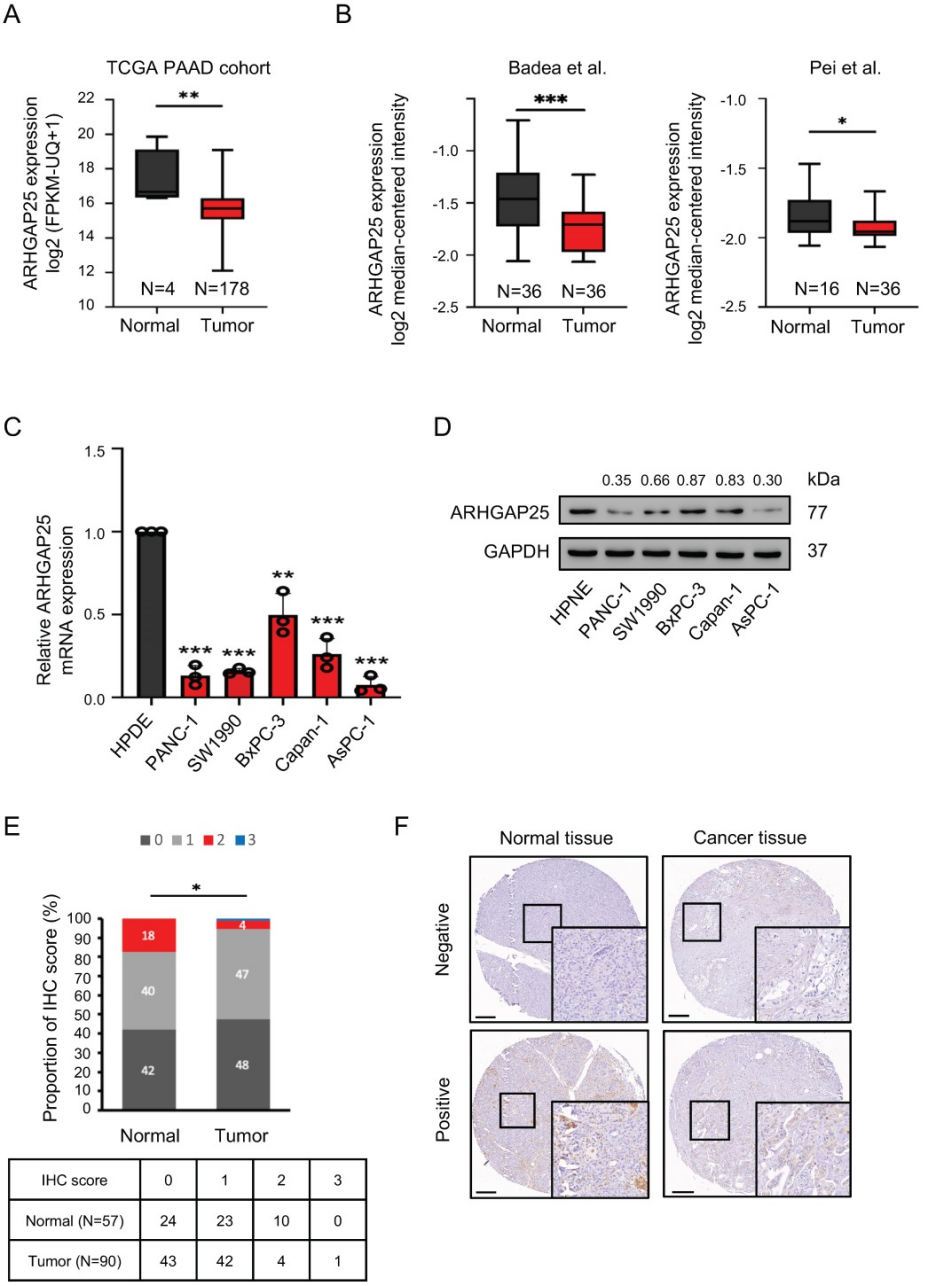
Rho GTPase activating protein 25 (ARHGAP25) expression was downregulated in pancreatic adenocarcinoma (PAAD). A, The gene expression of ARHGAP25 was reduced in PAAD tissues compared with that in normal tissues from the Cancer Genome Atlas (TCGA) database. B, ARHGAP25 gene expression was downregulated in PAAD tissues in GEO datasets (GSE15471 and GSE16515). C,D, mRNA expressions (n=3) (C) and protein levels (D) of ARHGAP25 were decreased in a panel of PAAD cell lines compared with that in Human pancreatic normal ductal epithelial cells (HPNE). E, The relative immunohistochemical (IHC) scores of ARHGAP25 was significantly lower in PAAD tissues than that of normal tissues. F, Representative IHC staining of ARHGAP25 in normal and tumor tissues. Scale bar: 200 μm. Data are presented as box plots with median and interquartile range (25% and 75%). *P < .05, **P < .01, ***P < .001.

We first observed that ARHGAP25 mRNA expression was lower in PAAD tissues than that in normal tissues, according to the data from the TCGA database (Figure 1A). Consistent results were observed in two GEO datasets (Figure 1B). To validate the findings of gene expression from public transcriptome datasets, we evaluated ARHGAP25 mRNA and protein levels in a panel of human PAAD cell lines by immunoblot analysis. Both ARHGAP25 mRNA and protein levels were consistently reduced in PAAD cells, including AsPC-1, PANC-1, BxPC-3, SW1990, and Capan-1 compared with the normal pancreatic cell line (HPNE) (Figure 1C, D). Furthermore, IHC staining revealed decreased ARHGAP25 expression levels in pancreatic tumor tissues compared with normal tissues (*P*=0.010, Figure 1E, F). As shown in table 1, we found that downregulated ARHGAP25 expression was significantly correlated with aggressive tumor characteristics, including tumor size (*P*=0.012) and T stage (*P*=0.036), but not with age, gender, tumor grade, and N stage.

### ARHGAP25 regulates PAAD proliferation in vitro and in vivo

**Figure 2:**
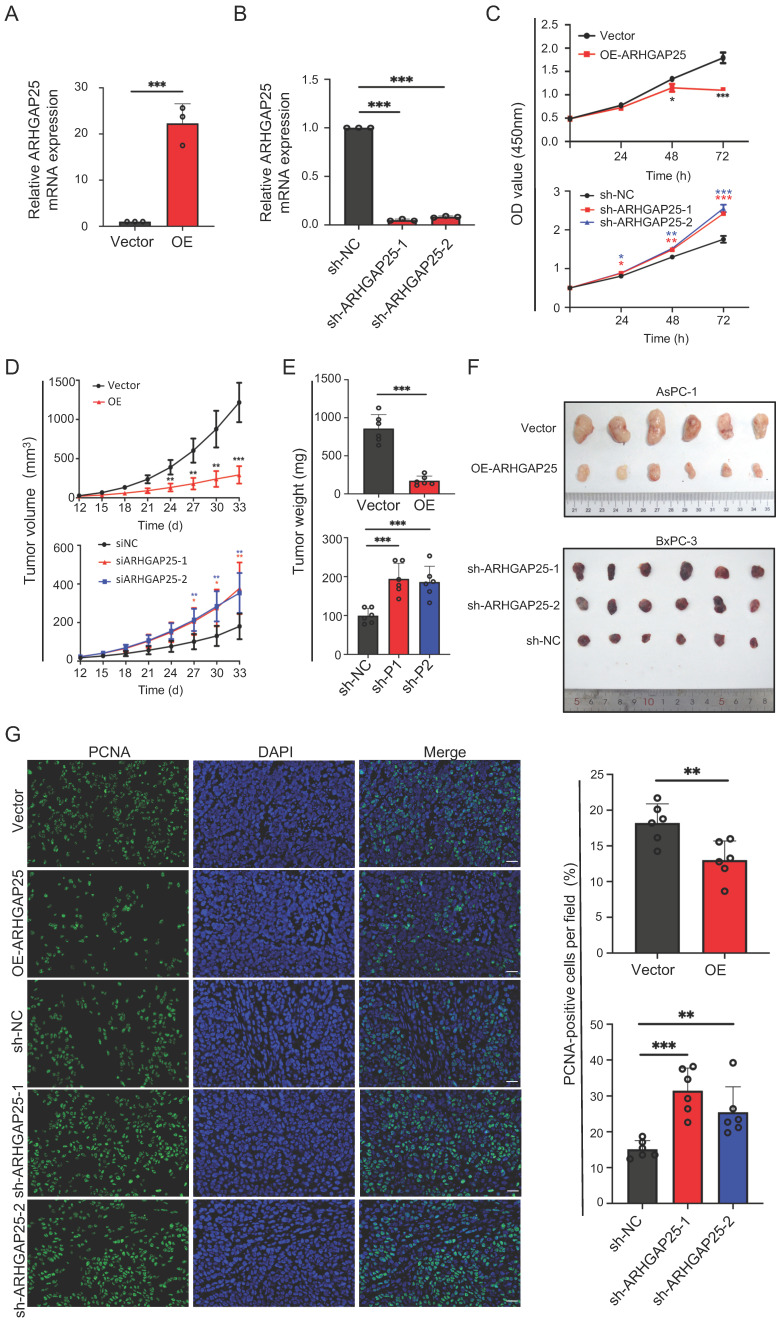
Rho GTPase activating protein 25 (ARHGAP25) inhibited pancreatic adenocarcinoma (PAAD) proliferation and tumorigenicity. A,B, Verification of ARHGAP25 overexpression and knockdown efficiency in PAAD cells revealed that the mRNA expression of ARHGAP25 was significantly higher in ARHGAP25-OE AsPC-1 cells (A) and lower in sh-ARHGAP25 BxPC-3 cells (B) compared to that in control cells (n=3 per group). C, CCK-8 assays revealed that the proliferation of ARHGAP25-OE AsPC-1 cells was significantly reduced, while that of sh-ARHGAP25 BxPC-3 cells was significantly increased (n=3 per group). D, Tumor growth curves showed that xenografts generated from ARHGAP25-OE AsPC-1 cells grew slower than those from ARHGAP25-NC cells (n=6). Conversely, xenografts generated from sh-ARHGAP25 BxPC-3 cells had increased growth rates compared with those from sh-NC cells (n=6). E,F, The weights and volumes of tumors in the ARHGAP25-OE group were significantly lower than those in the ARHGAP25-NC group (n= 6). The weights and volumes of tumors in the sh-ARHGAP25 group were significantly higher than those in the sh-NC group (n=6). G, Representative images (left panel) and quantification (right panel) of immunofluorescence staining with PCNA in xenograft tumors stably transfected as indicated were shown, Scale bar: 50 μm. Data are shown as the mean ± SD (n ≥ 3). *P < .05, **P < .01, ***P < .001.

Based on transcriptome and IHC data, we hypothesized that ARHGAP25 might function as a tumor suppressor of PAAD. To explore the effect of ARHGAP25 on PAAD cell proliferation, the AsPC-1 cell line, which has the lowest ARHGAP25 expression, was infected to stably overexpress ARHGAP25. The BxPC-3 cell line, which has the highest ARHGAP25 expression, was selected to silence ARHGAP25 through lentivirus infection. The efficiency of ARHGAP25 knockdown and overexpression was verified by qRT-PCR (Figure 2A,B). The results of CCK-8 assay showed that overexpression of ARHGAP25 inhibited AsPC-1 cell proliferation (Figure 2C). ARHGAP25 knockdown in BxPC-3 cells increased cell proliferation (Figure 2C).

**Figure 3:**
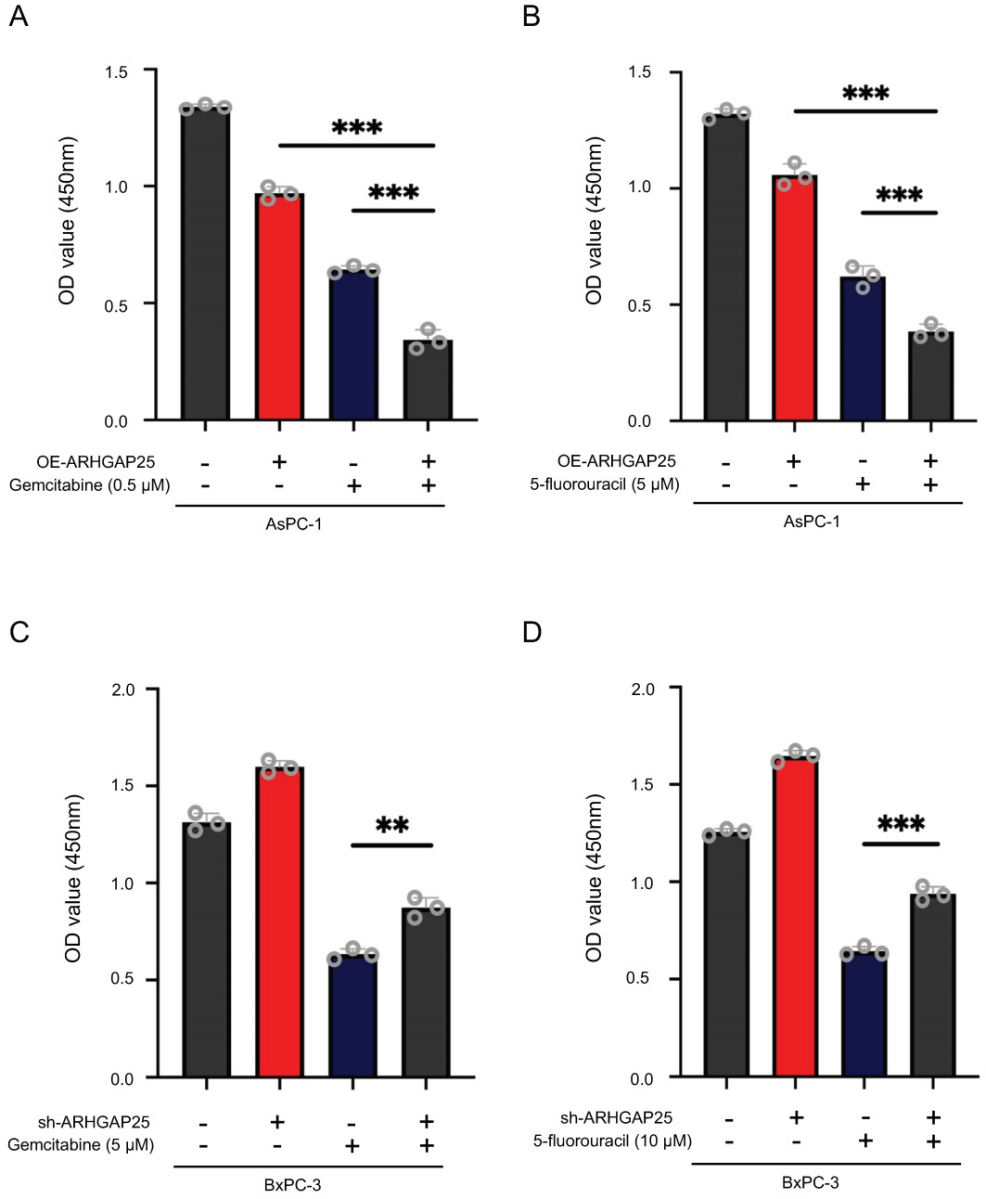
Rho GTPase activating protein 25 (ARHGAP25) attenuated cytotoxic effects of gemcitabine or 5-fluorouracil in pancreatic adenocarcinoma (PAAD) cells. A,B, ARHGAP25-overexpressing AsPC-1 cells were treated with gemcitabine (0.5µM) (A) and 5-fluorouracil (5 µM) (B) for 72 h. Upregulation of ARHGAP25 enhanced both gemcitabine and 5-fluorouracil cytotoxicity in AsPC-1 cells. C,D, ARHGAP25-knockdown BxPC-3 cells were treated with gemcitabine (5 µM) (C) and 5-fluorouracil (10 µM) (D) for 72 h. ARHGAP25 knockdown reduced the chemosensitizing effect of gemcitabine or 5-fluorouracil. The cell viability was measured by CCK-8 assay at 450nm. Data are shown as the mean ± SD. **P < .01, ***P < .001.

To further clarify whether AHRGAP25 could regulate PAAD tumorigenicity in vivo, subcutaneous xenograft mice models were established using AsPC-1 and BxPC-3 cell lines. As shown in Figure 2D, tumors generated from ARHGAP25-OE AsPC-1 cells grew slower and smaller than those generated from ARHGAP25-NC cells. On the other hand, ARHGAP25 knockdown promoted the growth of PAAD xenografts in nude mice. Consistently, the tumor weights and volumes in the ARHGAP25-OE group were significantly lower than those in the ARHGAP25-NC group, and vice versa in ARHGAP25 knockdown PAAD tumors (Figure 2E,F and S1,2). We further assessed the expression of proliferation markers, Ki-67 and proliferating cell nuclear antigen (PCNA), in xenograft sections using immunofluorescence. The expression of PCNA and Ki-67 was significantly reduced in xenografts overexpressing ARHGAP25 and increased in xenografts with ARHGAP25 knockdown (Figure 2G and S3). To investigate the effect of ARHGAP25 on chemotherapeutic cytotoxicity, we treated AsPC-1 and BxPC-3 cells with gemcitabine or 5-fluorouracil (5-FU) upon modulation of ARHGAP25. ARHGAP25-overexpressing AsPC-1 cells remarkably promoted cytotoxicity of gemcitabine and 5-FU (Figure 3A,B and S4). Moreover, ARHGAP25 silencing in BxPC-3 cells attenuated cytotoxic effects induced by gemcitabine or 5-FU (Figure 3C,D). Taken together, these results suggest that ARHGAP25 represses PAAD cell growth by inhibiting cell proliferation.

### ARHGAP25 regulates the AKT/mTOR signaling pathway in PAAD

**Figure 4:**
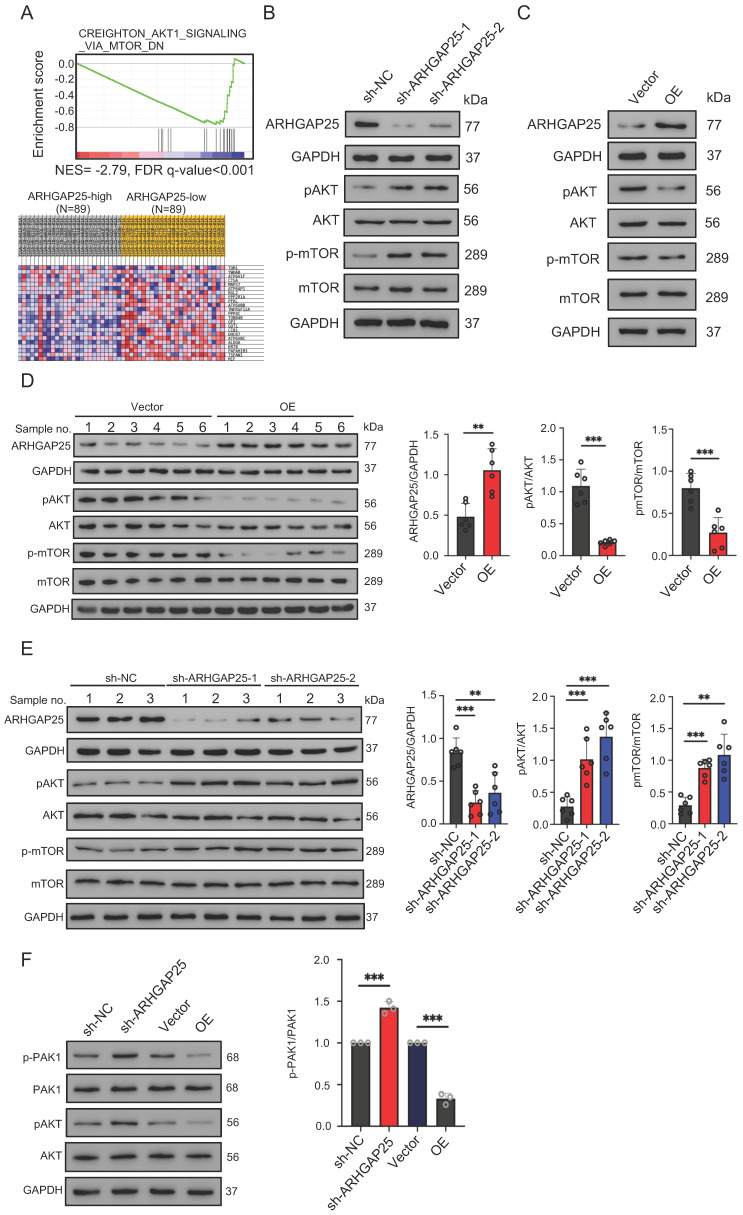
Rho GTPase activating protein 25 (ARHGAP25) inhibited AKT/mTOR signaling pathway. A, Gene set enrichment analysis (upper panel) showed the enrichment of AKT/mTOR signaling in PAAD tumors with downregulated ARHGAP25 expression. Differential expressions (lower panel) of the gene signature (CREIGHTON_AKT1_SIGNALING_VIA_MTOR_DN) are shown in the heatmap created by the GSEA software. B,C, Protein levels of ARHGAP25, total and phosphorylated AKT, and mTOR were examined by western blotting in PAAD cells. In sh-ARHGAP25 BxPC-3 cells, the expressions of p-AKT and p-mTOR were significantly increased (B). In ARHGAP25-overexpresisng AsPC-1 cells, the expressions of p-AKT and p-mTOR were significantly decreased (C). D,E, Western blotting was used to determine protein expression in xenografts. The expressions of p-AKT and p-mTOR were significantly decreased in the ARHGAP25-OE xenografts. (n=6) (D). Conversely, the expressions of p-AKT and p-mTOR were significantly increased in the xenografts with ARHGAP25 knockdown (n=6) (E). Data are shown as the mean ± SD. **P < .01, ***P < .001.

To gain insights into the biological processes involved in the ARHGAP25-induced phenotype change, we performed GSEA in the TCGA dataset. Among significantly upregulated biological processes in lower ARHGAP25 expression, AKT/mTOR signaling (CREIGHTON_AKT1_SIGNALING_VIA_MTOR_DN) was identified because of the important role of the PI3K/AKT/mTOR signaling pathway in PAAD development (Figure 4A). To determine whether the AKT/mTOR signaling is a key pathway for ARHGAP25-mediated regulation of PAAD cell proliferation, the expressions of total and phosphorylated AKT and mTOR were detected. ARHGAP25 overexpression decreased phosphorylated AKT and mTOR levels, while ARHGAP25 knockdown increased the levels of phosphorylated forms (Figure 4B,C) in PAAD cells. In line with these observations, western blot from xenograft tumor lysates confirmed that both phosphorylated AKT and mTOR levels were significantly lower in the ARHGAP25-OE xenografts than those in the ARHGAP25-NC group (Figure 4D). Phosphorylated AKT and mTOR levels were significantly increased in sh-ARHGAP25 xenografts compared to those in sh-NC xenografts (Figure 4E). These results indicated that ARHGAP25 regulates activation of the AKT/mTOR signaling in PAAD cells.

The mechanistic link between ARHGAP25 and AKT signaling remains unelucidated. As p21-activated kinase 1 (PAK1), a main downstream effector of Rac1 18, has been shown to activate AKT by serving as a scaffold 19 and ARHGAP25 has been shown to specifically inactivate the Rho family GTPase Rac1 20, we investigated whether ARHGAP25 inactivated AKT through PAK1 signaling. Both phosphorylated PAK1 and AKT levels were increased upon ARHGAP25 knockdown (Figure 4F). Overexpression of ARHGAP25 significantly reduced the phosphorylated PAK1 and AKT levels. These results suggest that ARHGAP25 inactivates AKT by negatively regulating Rac1-PAK1 signaling.

### ARHGAP25 regulates glycolysis in PAAD

**Figure 5:**
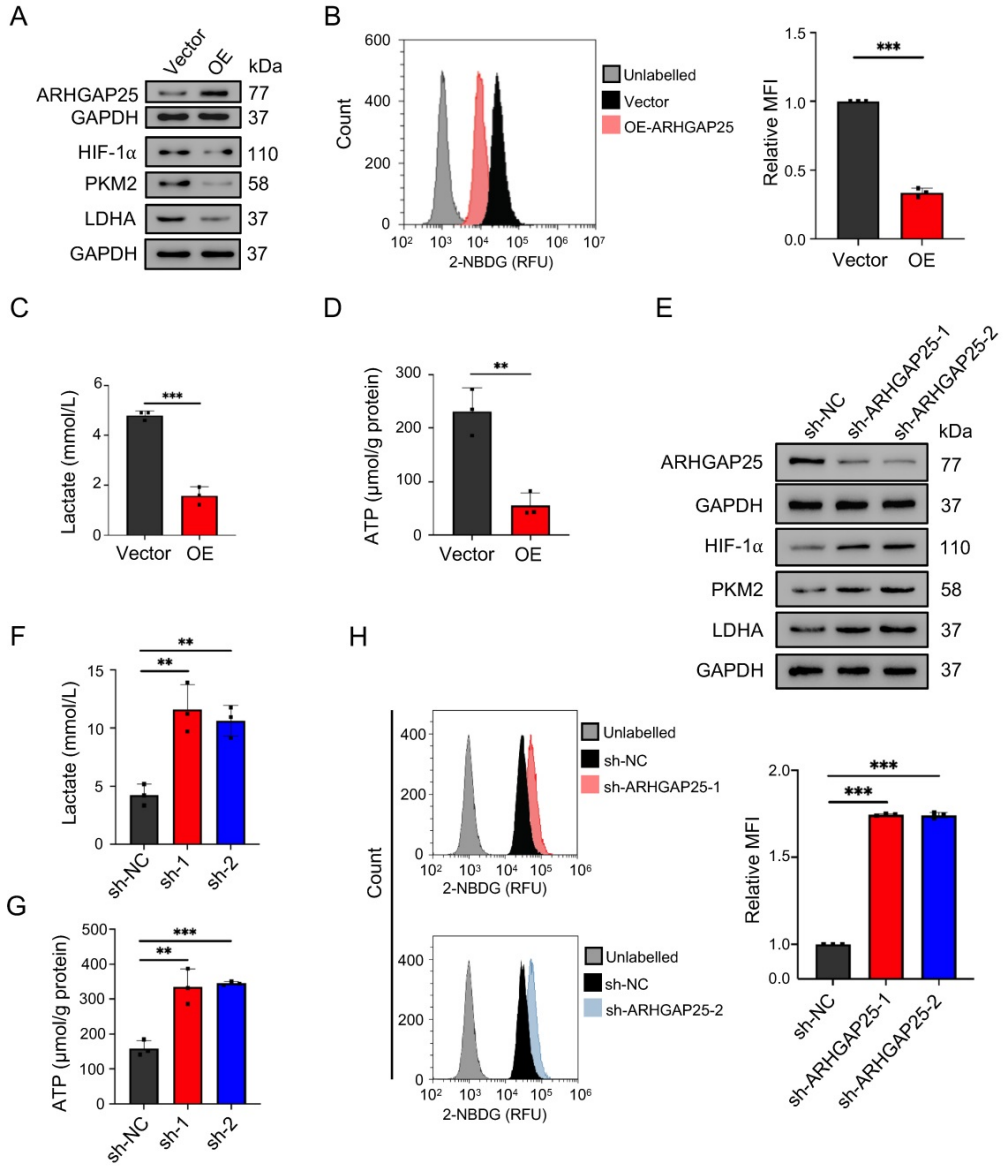
Rho GTPase activating protein 25 (ARHGAP25) suppressed glycolysis in PAAD in vitro. A, Protein levels of ARHGAP25, HIF-1α, PKM2, and LDHA were examined by western blotting in PAAD cells. In ARHGAP25-overexpresisng AsPC-1 cells, the expressions of HIF-1α, PKM2, and LDHA were significantly decreased. B, Glucose uptake was measured using the 2-NBDG uptake assay kit by flow cytometry. Overexpression of ARHGAP25 significantly decreased 2-NBDG uptake (n=3). C,D, Extracellular lactate levels and intracellular ATP levels were measured using the lactate assay kit and the ATP assay kit, respectively. Lactate production (C) and intracellular ATP levels (D) were significantly decreased in ARHGAP25-overexpresisng AsPC-1 cells (n=3 per group). E, In sh-ARHGAP25 BxPC-3 cells, the expressions of HIF-1α, PKM2, and LDHA were significantly increased. F,G, Lactate production (F) and intracellular ATP levels (G) were significantly increased in sh-ARHGAP25 BxPC-3 cells (n=3 per group). H, ARHGAP25 knockdown significantly increased 2-NBDG uptake (n=3). Data are shown as the mean ± SD. **P < .01, ***P < .001.

**Figure 6:**
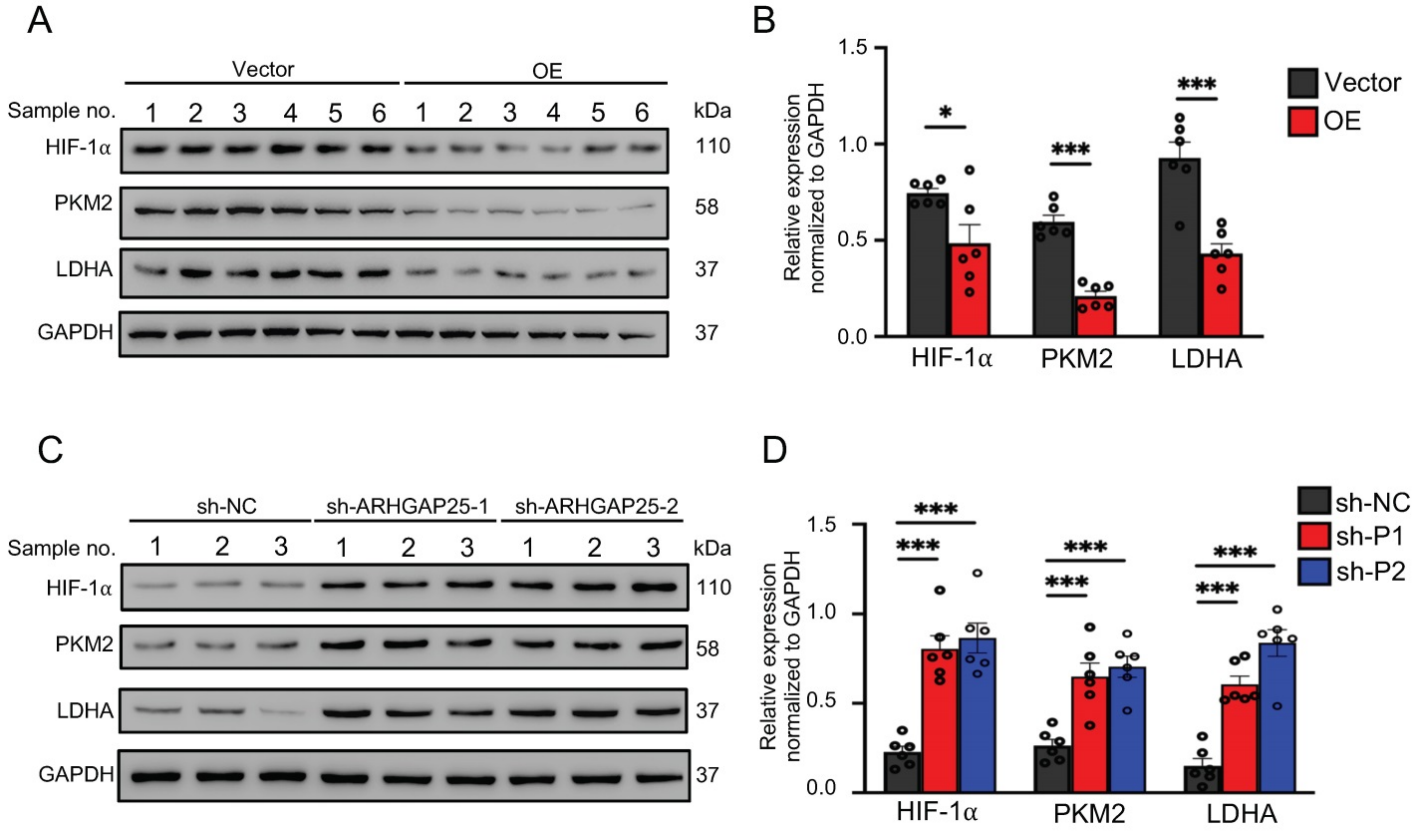
Rho GTPase activating protein 25 (ARHGAP25) suppressed glycolysis in PAAD in vivo. A,B, Western blotting was used to determine protein expression in xenografts. The expressions of HIF-1α, PKM2, and LDHA were significantly decreased in the ARHGAP25-OE xenografts (n=6). C,D, The expressions of HIF-1α, PKM2, and LDHA were significantly increased in the xenografts with ARHGAP25 knockdown (n=6). Data are shown as the mean ± SD. *P < .05, ***P < .001.

Activation of AKT/mTOR signaling promotes glycolysis in cancer cells 21. Thus, we further examined the effect of ARHGAP25 on glycolysis activity and hypoxia-inducible factor 1-alpha (HIF-1α), which is a crucial transcription factor involved in the upregulation of glucose transporter and genes associated with glycolysis. ARHGAP25 overexpression significantly decreased protein levels of HIF-1α, pyruvate kinase isozymes M2 (PKM2), and lactate dehydrogenase A (LDHA) in AsPC-1 cells (Figure 5A). Consistent with these observations, ARHGAP25 overexpression decreased glucose uptake, lactate production, and intracellular ATP levels (Figure 5B-D). In contrast, ARHGAP25 knockdown significantly increased protein levels of HIF-1α, PKM2, and LDHA in BxPC-3 cells (Figure 5E). The lactate production, intracellular ATP level, and glucose uptake were increased in BxPC-3 cells with ARHGAP25 knockdown (Figure 5F-H). To further confirm these in vitro findings, we detected protein levels in xenograft lysates using western blotting. Protein levels of HIF-1α, PKM2, and LDHA were significantly decreased in ARHGAP25-OE xenografts (Figure 6A,B), while increased in sh-ARHGAP25 xenografts (Figure 6C,D). Taken together, these results indicated that ARHGAP25 could regulate HIF-1α-mediated glycolysis in PAAD.

### ARHGAP25 regulates glycolysis and PAAD cell proliferation by suppressing AKT/mTOR signaling pathway in PAAD

**Figure 7:**
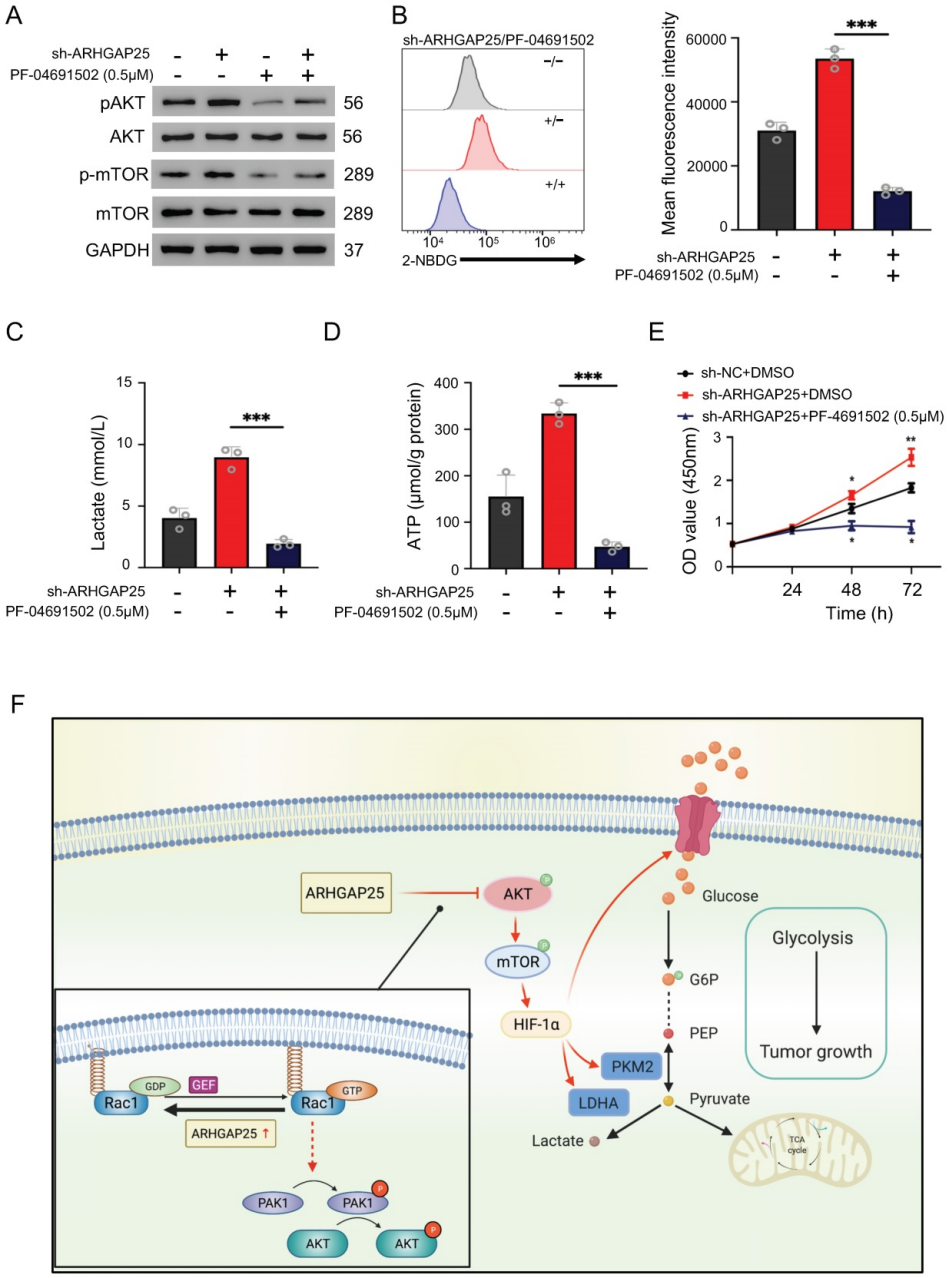
Silencing of Rho GTPase activating protein 25 (ARHGAP25) promoted glycolysis and proliferation by activating AKT/mTOR signaling in PAAD cells. A, Western blot analysis showed that PI3K and mTOR inhibitor PF-04691502 reduced activation of AKT/mTOR signaling induced by ARHGAP25 knockdown. B, Increased glucose uptake in sh-ARHGAP25 BxPC-3 cells was remarkably reduced upon PF-04691502 treatment (n=3). C,D, Upregulated lactate production (C) and intracellular ATP level (D) upon ARHGAP25 knockdown were abolished by PF-04691502 in BxPC-3 cells (n=3 per group). E, The effect of ARHGAP25 knockdown on promoting cell growth was remarkably abrogated by PF-04691502 (n=3). F, The schematic diagram showed the mechanism of ARHGAP25 regulating glycolysis and cell growth through AKT/mTOR signaling in PAAD.

To further confirm whether ARHGAP25 regulates glycolysis and PAAD cell proliferation via the AKT/mTOR pathway, we treated sh-ARHGAP25 BxPC-3 cells with PF-04691502, a dual inhibitor of PI3K and mTOR. PF-04691502 treatment reduced the increased protein levels of phosphorylated p-AKT and p-mTOR upon ARHGAP25 knockdown (Figure 7A). Upregulated glucose uptake, lactate production, and intracellular ATP level in sh-ARHGAP25 BxPC-3 cells were largely abolished by PF-04691502 (Figure 7B-D). Moreover, augmented PAAD cell proliferation due to ARHGAP25 knockdown was impeded upon PF-04691502 treatment (Figure 7E). These results indicated that loss of ARHGAP25 promotes glycolysis and cell proliferation of PAAD via the AKT/mTOR signaling pathway (Figure 7F).

## DISCUSSION

The RhoGAP gene family consists of at least 60 members, which regulate cellular motility, proliferation and differentiation via regulating the Rho GTPase family 22. ARHGAP25 is homologous to ARHGAP24 and ARHGAP22, which are RhoGAPs specific for the small Rho GTPase Rac, leading to its inactivation. ARHGAP24 has been shown to act as a tumor suppressor in renal cell carcinoma and lung cancer 7, 23. Recent studies also found that the abrogation of ARHGAP22 promotes tumor cell motility 24. Consistently, ARHGAP25 has been shown to suppress lung cancer cell growth 15. However, its expression pattern and biologic significance in PAAD remained unclear. Here, we demonstrated that ARHGAP25 protein and mRNA expression were significantly decreased in PAAD. Therefore, we further investigated the role of ARHGAP25 in PAAD.

Previous studies have shown that ARHGAP25 is involved in cell proliferation and invasion in lung cancer, breast cancer, and rhabdomyosarcoma cells 14, 15, 25. Thuault et al. found that ARHGAP25 expression is upregulated in alveolar rhabdomyosarcoma (ARMS). They demonstrated that ROCKII suppresses Rac activity via ARHGAP25, reducing cell spreading using an ARMS-derived Rh4 cell line. These results indicate the suppressive role of ARHGAP25 in ARMS cell invasion by inhibiting Rac activity 14. Xu et al. showed that the expression of ARHGAP25 in lung tumor samples was downregulated compared with non-cancerous lung tissues. Furthermore, they disclosed the tumor suppressive role of ARHGAP25 in lung cancer cell growth, migration, and invasion through Wnt/β-catenin signaling 15. Our results also demonstrated that ARHGAP25 inhibits PAAD cell proliferation and tumorigenicity in vitro and in vivo. In line with the GSEA bioinformatics analysis, we found that overexpression of ARHGAP25 reduces PAAD cell proliferation by suppressing AKT/mTOR signaling.

The mechanism underlying the regulation between ARHGAP25 and AKT/mTOR signaling remained to be elucidated. p21-activated kinase 1 (PAK1), an effector of Rac, was shown to promote AKT phosphorylation by serving as a scaffold to enhance the interaction between AKT and 3-Phosphoinositide-dependent kinase 1 (PDK1) 19. We did demonstrate that both phosphorylated PAK1 and AKT were suppressed by the upregulation of ARHGAP25. Moreover, ARHGAP25 might interact with AKT. Two RhoGAPs, DLC1 and ARHGAP22, are phosphorylated by AKT 26, 27. Of note, DLC1 exerts RhoGAP-independent signaling via phosphorylation by other interacting proteins 28. Whether ARHGAP25 interacts with AKT and exerts RhoGAP-independent effects need further in-depth study.

Aberrant upregulation of aerobic glycolysis promoted by PKM2 is critical for tumor cell growth in many cancer types, including PAAD 29. Moreover, mTOR upregulates PKM2 expression through induction of HIF-1α under normoxic condition 30. Mechanistically, activation of mTOR increases HIF-1α protein level through upregulation of 5'cap-dependent translation 31. Therefore, the inhibition of glycolysis by ARHGAP25 overexpression in our study may be through suppressing the mTOR/HIF-1α/PKM2 signaling pathway. Interestingly, PI3K activates Rac to mobilize aldolase, a glycolytic enzyme, from the F-actin, promoting glycolysis in an AKT-independent manner 32. Whether ARHGAP25 may regulate glycolysis through the inactivation of Rac needs further investigation.

In conclusion, the present study revealed that ARHGAP25 acted as a tumor suppressor regulating cell growth and suppressed glycolysis through AKT/mTOR signaling in PAAD. These results suggest that ARHGAP25 is a potential therapeutic target to repress PAAD progression.

## Supplementary Material

Supplementary figures and tables.

## Figures and Tables

**Table 1:** Correlation between ARHGAP25 expression and clinicopathological characteristics.

Characteristics	Total	ARHGAP25 expression		*P*-value
		Negative (N= 43)	Positive (N=47)	
**Age, y**				0.148
≤60	41	23	18	
>60	49	20	29	
**Gender**				0.753
Male	58	27	31	
Female	32	16	16	
**Grade**				0.960
Well	7	3	4	
Moderate	64	31	33	
Poor	19	9	10	
**Tumor size, cm**				**0.012**
<5	54	20	34	
≥5	36	23	13	
**T stage**				
T1-2	73	31	42	**0.036**
T3	17	12	5	
N stage*				0.629
N0	46	20	26	
N1	39	19	20	

Note: *N stage assessment of 85 patients. *P*-values reflect the relationship between ARHGAP25 expression and clinicopathological characteristics with chi-square test. *P* < 0.05 was considered statistically significant and shown in bold. Abbreviations: ARHGAP25, Rho GTPase activating protein 25.

## Abbreviations

AKT: protein kinase B
ARHGAP: Rho GTPase activating protein
ATCC: American Type Culture Collection
DLC1: deleted in liver cancer 1
GEO: gene expression omnibus
GSEA: gene set enrichment analysis
HIF-1α: hypoxia-inducible factor 1-alpha
HPNE: human normal pancreatic duct epithelial
HRP: horseradish peroxidase
IHC: Immunohistochemistry
LDHA: lactate dehydrogenase A
MOI: multiplicity of infections
mTOR: mammalian target of rapamycin
NC: empty vector
OE: overexpressing vector
PAAD: pancreatic adenocarcinoma
PCNA: proliferating cell nuclear antigen
PKM2: Pyruvate kinase isozymes M2
qRT-PCR: quantitative real-time reverse transcription-polymerase chain reaction
RhoGAP: Rho GTPase activating protein
sh-NC: lentiviral plasmid expressing negative control short hairpin RNA
TCGA: The Cancer Genome Atlas
TMA: Tissue microarray
