# Supplementary material for: ARHGAP25 Inhibits Pancreatic Adenocarcinoma Growth by Suppressing Glycolysis via AKT/mTOR Pathway
Source: Int J Biol Sci. 2021 Apr 24;17(7):1808–20. doi: 10.7150/ijbs.55919 (PMC8120455; doi:10.7150/ijbs.55919)
Supplement: Supplementary file 1 — Supplementary figures and tables. [file ijbsv17p1808s1.pdf]

Table S1 List of short hairpin RNA sequences against ARHGAP25 and primer sequences for cloning the coding region of ARHGAP25.

pLKO.1 shARHGAP25-1	Forward	CCGGTCCATCCTTCCTCGTGACAACTCGAGTT GTCACGAGGAAGGATGGTTTTTG
	Reverse	AATTCAAAAACCATCCTTCCTCGTGACAACTC GAGTTGTCACGAGGAAGGATGGA
pLKO.1 shARHGAP25-2	Forward	CCGGTGGACTCAAACACTCCCTAACTCGAGTT AGGGAGTGTTTGAGTCCTTTTTG
	Reverse	AATTCAAAAAGGACTCAAACACTCCCTAACTC GAGTTAGGGAGTGTTTGAGTCCA
Primers for cloning	Forward	5’ -CGGAATTCATGTCCCTAAAATTGCCAAGG-3’
	Reverse	5’-CGGGATCCTTAAGCCTCGGTCTTG-3’

Figure S1. Mice were inoculated subcutaneously with AsPC-1 cells stably transfected with vector control or OE-ARHGAP25 (n=6 in each group).

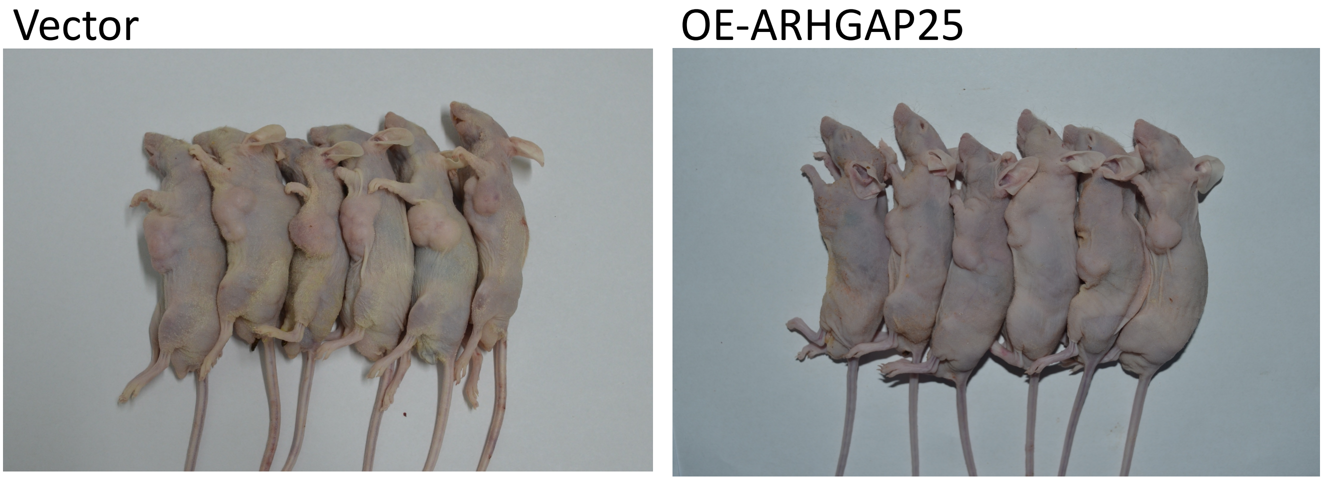

Figure S2. Mice were inoculated subcutaneously with BxPC-3 cells stably transfected with vector control or sh-ARHGAP25 (n=6 in each group).

sh-NC

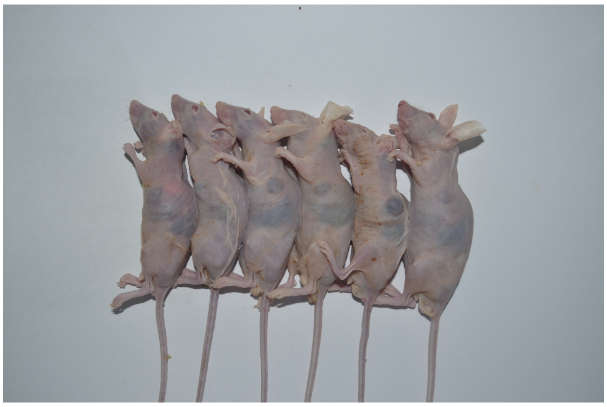

sh-ARHGAP25-1

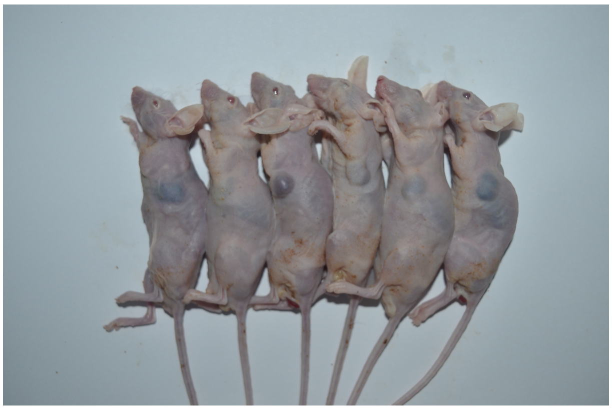

sh-ARHGAP25-2

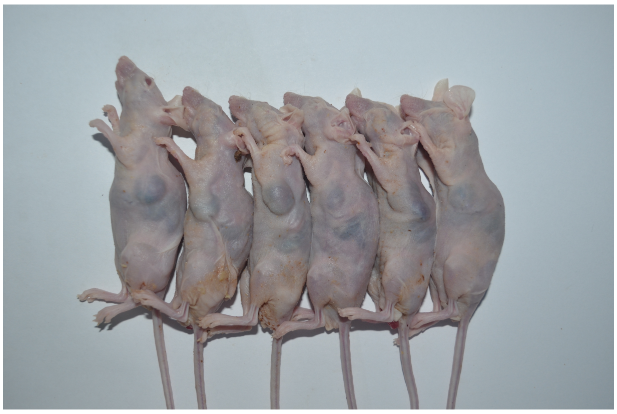

Figure S3. Representative images (left panel) and quantification (right panel) of immunofluorescence staining with Ki-67 in xenograft tumors stably transfected as indicated were shown, Scale bar: 50 μm. Data are shown as the mean ± SD (n ≥ 3). **P < .01, ***P < .001.

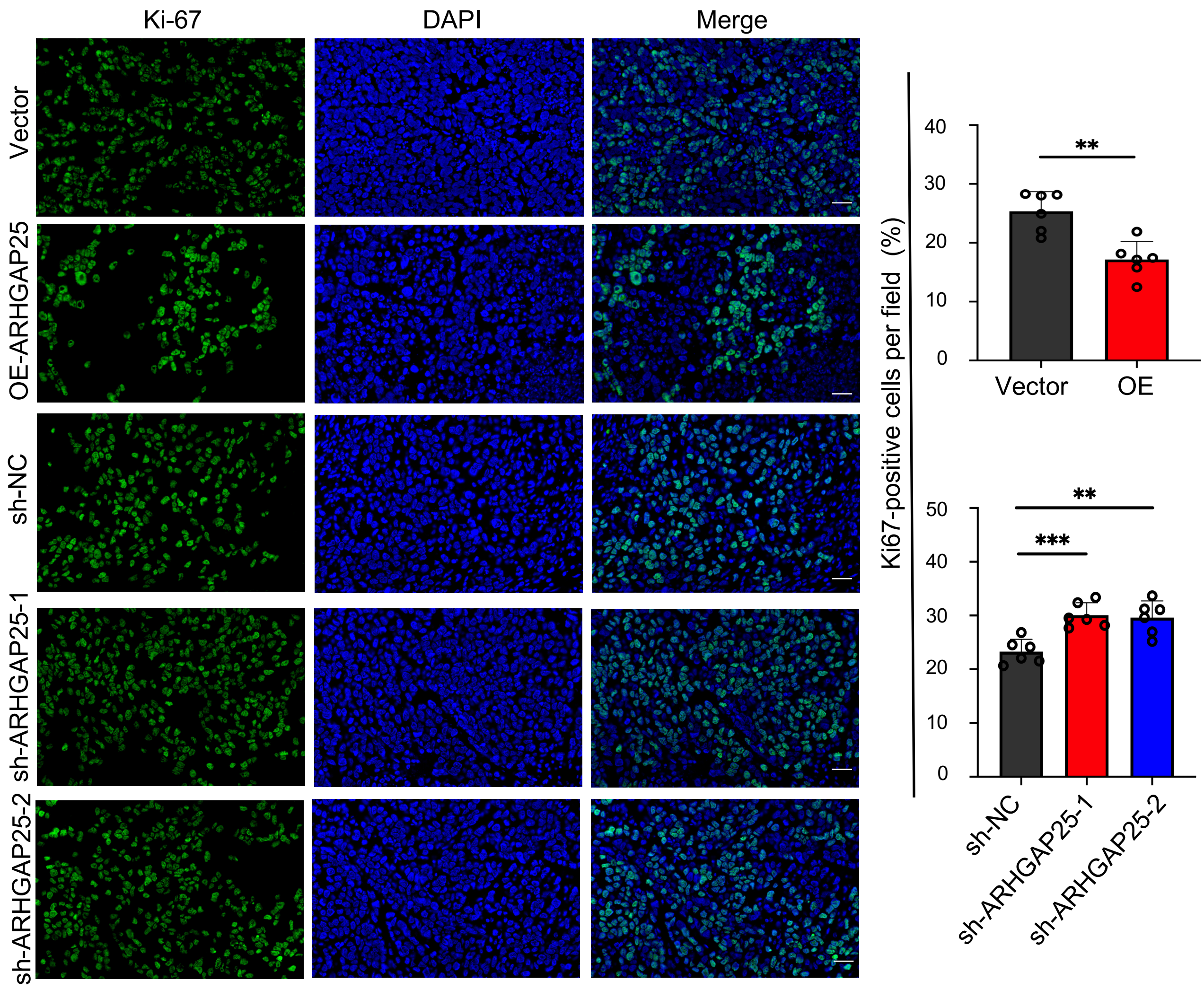

Figure S4. Both AsPC-1 and BxPC-3 cells were treated with a serial concentration of gemcitabine or 5-fluorouracil for 72 h. The relative cell viability was evaluated using the CCK-8 assay. The IC50 values of gemcitabine or 5-fluorouacil were also shown.

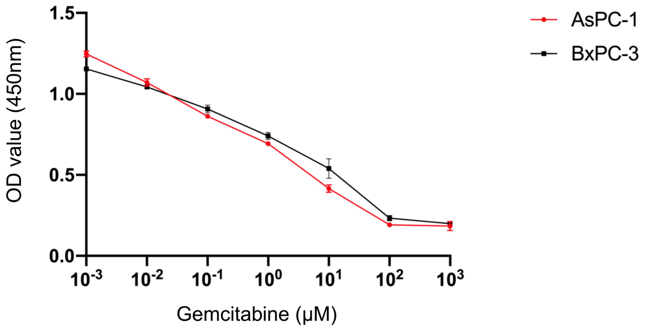

Cell line	AsPC-1	BxPC-3
IC50 GEM (μM)	0.504	3.048

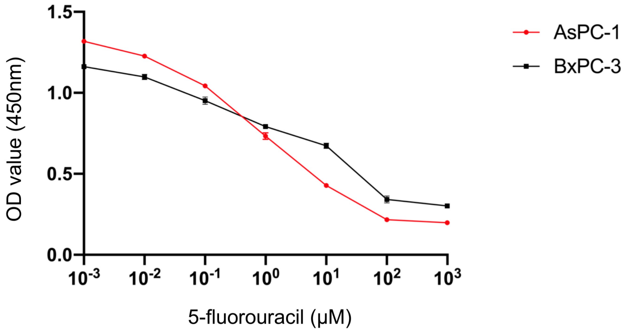

Cell line	AsPC-1	BxPC-3
IC50 5-FU (μM)	5.778	7.846
